# Seroprevalence and Current Infections of Canine Vector-Borne Diseases in Costa Rica

**DOI:** 10.3389/fvets.2019.00164

**Published:** 2019-06-04

**Authors:** Andrea Springer, Víctor M. Montenegro, Sabine Schicht, Majda Globokar Vrohvec, Nikola Pantchev, Jörg Balzer, Christina Strube

**Affiliations:** ^1^Centre for Infection Medicine, Institute for Parasitology, University of Veterinary Medicine Hannover, Hanover, Germany; ^2^Laboratory of Parasitology, School of Veterinary Medicine, National University of Costa Rica, Campus Benjamín Núñez, Heredia, Costa Rica; ^3^IDEXX Laboratories, Ludwigsburg, Germany

**Keywords:** vector-borne diseases, tick-borne diseases, zoonoses, *Rickettsia* spp., *Ehrlichia* spp., *Anaplasma* spp., *Babesia* spp., Central America

## Abstract

Domestic dogs may carry several vector-borne pathogens, including zoonotic agents, especially in tropical regions like Central America. The epidemiology of these pathogens is prone to change due to urbanization, trade and travel as well as climate change, necessitating repeated monitoring. This study aims to present a comprehensive picture of canine vector-borne diseases in Costa Rica, combining data on seroprevalence with molecular species identification of the causative pathogens. In this survey, 294 dogs from all seven provinces of Costa Rica were included. After a clinical examination, diagnostic blood samples were analyzed with regard to packed cell volume (PCV) and presence of microfilaria. Serum samples were tested for antibodies against *Ehrlichia* spp., *Anaplasma* spp., *Babesia* spp., *Borrelia burgdorferi* sensu lato (s.l.) as well as antigen of *Dirofilaria immitis*. Seropositive and microfilaremic blood samples were analyzed by PCR to detect current infections and identify the pathogen species. Overall, 45.24% (133/294, 95% CI: 39.45–51.11%) of dogs were seropositive for at least one of the tested pathogens. Seroprevalence was highest for *Ehrlichia* spp. (39.46%, 116/294, 95% CI: 33.83–45.29%), followed by *Babesia* spp. (23.13%, 68/294, 95% CI: 18.43–28.38%), *Anaplasma* spp. (13.27%, 39/294, 95% CI: 9.61–17.69%), and *B. burgdorferi* s.l. (0.34%, 1/294, 95% CI: 0.01–1.88%). Generalized linear mixed models indicated a significant association of *Ehrlichia*-, *Anaplasma*- and *Babesia*-seropositivity, as well as a significant effect of age and breed on *Ehrlichia*-seropositivity. Furthermore, a statistically significant negative effect of *Ehrlichia*-, *Anaplasma*-, and *Babesia*-seropositivity on PCV was found. Regarding current infections, *Ehrlichia canis* DNA was detected in 51.72% (60/116, 95% CI: 42.26–61.10%) of *Ehrlichia*-seropositive dogs, while *Ehrlichia ewingii* and *Ehrlichia chaffeensis* were not detected. Furthermore, 10.26% (4/39, 95% CI: 2.87–24.22%) of *Anaplasma*-seropositive dogs were coinfected with *Anaplasma phagocytophilum* and *Anaplasma platys*, while one animal (2.56%, 95% CI: 0.65–13.48%) was infected with *A. phagocytophilum* only. Among *Babesia*-seropositive dogs, *Babesia vogeli* and *Hepatozoon canis* were detected in one animal each (1.47%, 1/68, 95% CI: 0.04–7.92%). *Dirofilaria immitis* antigen was detected in 4.42% (13/294, 95% CI: 2.38–7.44%) of dogs. In microfilaremic animals, *D. immitis* as well as *Acanthocheilonema reconditum* infections were identified. This survey demonstrates that canine vector-borne pathogens, including zoonotic agents like *A. phagocytophilum* and *D. immitis*, are widespread in Costa Rica. Thus, protection of dogs from disease-transmitting vectors is recommended from an animal welfare as well as public health perspective.

## Introduction

Vector-borne diseases, including babesiosis, ehrlichiosis, anaplasmosis and dirofilariosis, may severely compromise canine health. Although often asymptomatic, these infections may lead to life-threatening symptoms such as anemia and thrombocytopenia with increased bleeding tendency, for example, as well as to a variety of unspecific symptoms (1). Furthermore, in the chronic stage of infection, ehrlichiosis, borreliosis, babesiosis and dirofilariosis, among other canine vector-borne diseases, can lead to glomerulopathies with proteinuria in dogs (2). In addition, some of these infections, e.g., dirofilariosis and granulocytic anaplasmosis, represent zoonoses (3). Their presence in dogs may thus indicate a health risk for humans.

Vector-borne diseases are often widespread in tropical regions, including Central America, due to optimal conditions for vectors such as mosquitoes and ticks (4). Tick infestation of dogs is common in this region and mainly involves the brown dog tick, *Rhipicephalus sanguineus* sensu lato (s.l.) (5–7), which is a competent vector for *Ehrlichia canis, Hepatozoon canis, Babesia vogeli*, and different *Rickettsia* species, among others (8). Although different clades of *R. sanguineus* s.l. may vary in their vector capacity, genetic studies have revealed that *R. sanguineus* s.l. specimens from Central America belong to the so-called “tropical lineage” with proven vector capacity for *E. canis* (9). Accordingly, previous surveys of canine vector-borne diseases in Central America have revealed high levels of exposure to *Ehrlichia* spp. (7, 10), followed by *Anaplasma* spp. (10, 11). Both *Anaplasma platys*, the causative agent of canine cyclic thrombocytopenia, and *Anaplasma phagocytophilum*, causing zoonotic granulocytic anaplasmosis, are present in the region (10, 12, 13). Additionally, *B. vogeli* as well as *Babesia gibsoni* have been detected by PCR in dogs from certain locations in Costa Rica and Nicaragua (14–16), but large-scale serological surveys on canine babesiosis in Central America are lacking. In contrast, the region does not appear to be endemic for *Borrelia burgdorferi* s.l., the causative agent of Lyme borreliosis, as infections have been detected only sporadically (11). Mosquito-borne *Dirofilaria immitis* infections have so far mainly been found in a regional pattern along the Pacific Coast of Costa Rica (11, 17), in accordance with studies from Mexico demonstrating higher *D. immitis* prevalences along shorelines (18).

However, the epidemiology of vector-borne diseases is prone to change under the influence of urbanization, changing land use patterns, human trade and travel as well as climate change (4), necessitating repeated monitoring. This study aims to present a comprehensive picture of canine vector-borne diseases in Costa Rica, combining data on the seroprevalence of *Ehrlichia* spp., *Anaplasma* spp., *Babesia* spp., *B. burgdorferi* s.l., and *D. immitis* among 294 dogs sampled in 2014 with molecular species identification of the causative pathogens.

## Methods

### Clinical Examination and Sampling of Dogs

From March to August 2014, 294 dogs were sampled at 21 different locations in Costa Rica, distributed over all seven provinces. The dogs were presented at randomly selected veterinary clinics for varying reasons, e.g., vaccinations, health checks or curative consultations. Dogs from an animal shelter were only included at one location (San Rafael de Heredia, *N* = 30). Only dogs more than 6 months of age, which had not been treated with ivermectin during the last 6 months nor with doxycycline during the last 12 months, were included in the study, and consent of the owner to use surplus samples for further analyses was obtained. The dogs received a clinical examination and sex, age and breed were noted. Diagnostic blood samples were taken from the cephalic or jugular vein and collected into serum and EDTA tubes. Packed cell volume (PCV) was determined by glass capillary centrifugation of EDTA blood. Remaining EDTA blood and serum was stored at −20°C until shipping to Germany on dry ice for further analyses.

### Screening of Blood Samples for Vector-Borne Pathogens

Serum samples were tested for antibodies against *Anaplasma* spp., *Ehrlichia* spp. and *B. burgdorferi* s.l., as well as antigen of *D. immitis* by use of a commercial rapid ELISA (SNAP®4DXPlus®, IDEXX Laboratories Inc., Westbrook, ME, USA). Sensitivity and specificity of this test system are as follows: 93.2 and 99.2% for *A. phagocytophilum*, 89.2 and 99.2% for *A. platys*, 96.7 and 98.8% for *B. burgdorferi* s.l., 97.8 and 92.3% for *E. canis*, and for *D. immitis* 98.9 and 99.3% (19). Regarding *Ehrlichia* spp., cross-reactivity of the *E. canis* antigen (peptides from p30 and p30-1 outer membrane proteins) with anti-*Ehrlichia chaffeensis* has been shown (19); the device additionally detects antibodies to *Ehrlichia ewingii* (peptide derived from p28 outer surface protein family) Furthermore, cross-reactivity between *A. phagocytophilum* and *A. platys* has also been demonstrated (peptide from the major surface protein p44/MSP2) (20). Thus, we refer to *Anaplasma* spp. and *Ehrlichia* spp. as results in the present study.

To detect IgG antibodies against *Babesia* spp., a commercial ELISA test kit was used (Babesia ELISA DOG, afosa GmbH, Blankenfelde-Mahlow, Germany). The reference range of the test score is negative (<14), borderline at 14–19 and positive [>19; for further details see (21)]. According to the manufacturer, sensitivity and specificity of this test for *B. canis* compared with the indirect immunofluorescence assay are 91.6 and 95.4%, respectively. However, cross-reactions with other *Babesia* spp. (*B. vogeli* and *B. gibsoni*) as well as the related piroplasm *Rangelia vitalii* occur (22, 23), and we refer to antibodies against *Babesia* spp. accordingly.

To determine which *Anaplasma*- and *Ehrlichia*-seropositive dogs were currently infected (as defined by DNA detection) with *E. canis, A. phagocytophilum* and *A. platys*, respectively, species-specific PCRs were carried out as described previously (10). Briefly, DNA was isolated from blood samples using the Nucleospin® 8 Blood Kit (Macherey-Nagel GmbH & Co. KG, Düren, Germany). To detect *A. phagocytophilum*, a nested PCR targeting a 546 bp fragment of the 16S rRNA was carried out using primers ge3a and ge10r in a first and ge9f and ge2 in a second PCR round (24). For *A. platys*, a 678 bp fragment of the 16S rRNA gene was targeted by a nested PCR using primer sets 8F and 1448R for a first and EHR16SR and PLATYS for a second PCR round (25). For detection of *E. canis*, a 389 bp fragment of the 16S rRNA gene was targeted by nested PCR using primer pairs ECC and ECB in a first and ECAN5 and HE3 in a second PCR round (26, 27). PCR products were visualized by gel electrophoresis on 2% agarose gels. Furthermore, all samples seropositive for *Ehrlichia* spp. were additionally subjected to quantitative real-time PCR for detection of *E. canis, E. chaffeensis* and *E. ewingii* DNA as described previously (22).

To determine whether *Babesia*-seropositive dogs were currently infected, a genus-specific, semi-nested PCR targeting a 350 bp fragment of the 18S rRNA gene was carried out, using primers BJ1 and BN2 (28) in the first round, and BJ1 and PIRO-B (29) in the second round. The 25 μl reaction volume contained 2.5 μl DreamTaq® PCR Mastermix (Thermo Fisher Scientific Inc., Waltham, MA, USA), 0.5 μl of dNTPs (10 mM each), 0.5 μl of each primer (10 μM), 15.5 μl deionized water and 5 μl template DNA. In the second PCR, 1 μl of PCR-product from the first round was included as template, and the amount of water adjusted accordingly. For each round, the following thermoprofile was carried out in a peqSTAR thermocycler (peqlab Biotechnologie GmbH, Erlangen, Germany): initial denaturation at 95°C for 3 min, followed by 40 cycles at 94°C for 30 s, 55°C for 30 s and 72°C for 1 min, and final extension at 72°C for 10 min. Amplicons of the correct size were sequenced with primer BJ1 at a commercial sequencing laboratory (Microsynth Seqlab Sequence Laboratories, Göttingen, Germany). Present infections with *B. burgdorferi* s.l. were not further investigated due to low seroprevalence.

Additionally, buffy coat of all dogs was investigated microscopically for presence of microfilariae. Samples which contained microfilariae in buffy coat were subjected to a PCR targeting the internal transcribed spacer (ITS) 1-5.8S rDNA-ITS2 complex by use of primers NC2 and NC5 (30) as described previously (10), and amplicons were custom-sequenced in both directions (Microsynth Seqlab Sequence Laboratories, Göttingen, Germany). Obtained sequences were assembled using Clone Manager 9 Professional Edition (Scientific and Educational Software, Denver, CO, USA) and compared with sequences deposited in the GenBank database of the National Centre for Biotechnology Information (NCBI) using the Basic Local Alignment Search Tool (BLAST).

### Statistical Analyses

Statistical analyses were conducted in R v. 3.5.0 (31). To assess which factors influenced the likelihood of being seropositive for *Ehrlichia* spp., *Anaplasma* spp. and *Babesia* spp., respectively, generalized linear mixed models (GLMMs) with binomial error structure and logit-link function were constructed [function “glmer,” package “lme4” (32)]. The following predictor variables were included as fixed factors: dog sex, dog age (years), dog breed (dichotomized as with breed/mongrel), and whether the sampling location was a city, a western or eastern coastal area, a rural area at high altitude [defined as ≥1,000 m above sea level (asl)] or low altitude (<1,000 m asl). To examine associations between seropositivity for the different pathogens, test results for *Babesia* spp. and *Anaplasma* spp. were included as fixed factors in the model for *Ehrlichia* spp., and vice versa. The location of sampling was included as a random factor. Multiple comparisons for factors with more than two levels were carried out using the function “glht” [package “multcomp” (33)], with Tukey HSD single-step *P*-value adjustment.

To examine the relationship between seropositivity and packed cell volume (PCV), we used a linear mixed model (LMM, package “lme4”), including presence of antibodies against *Ehrlichia, Anaplasma* and *Babesia* spp. and antigen of *D. immitis* as fixed factors, and location of sampling as a random factor. Because animal age and sex may affect PCV (34), these variables were included as additional fixed factors. Initially, interactions between all four pathogens were included, and were removed if not significant. LMM fit was assessed by inspecting normality and homogeneity of model residuals. Full models were compared to null models containing only the random factor in a likelihood ratio test (R function “anova,” method = “chisq”).

The ratio of animals displaying clinical symptoms compatible with the investigated vector-borne diseases (i.e., anorexia, apathy, fever, lymphadenopathy, pale mucous membranes, epistaxis, petechia and/or cough) was compared between seronegative animals and animals seropositive for at least one of the tested pathogens using a Chi-square-test.

## Results

### Clinical Presentation of Dogs

In total, 294 dogs were included in the study (21–73 per province, Table 1), comprising 215 mongrels and 79 dogs with a breed. In the clinical examination, 25.51% (75/294) of dogs were infested with ticks, while 25.17% (74/294) showed flea infestation. Pale mucous membranes were noted in 22.11% (65/294) of dogs, two of these (0.68%) additionally showed petechial bleeding. Three further dogs (1.02%) showed epistaxis. Furthermore, 3.74% (11/294) of dogs presented with fever, 3.40% (10/294) with apathy, 2.04% (6/294) with anorexia, and 1.02% (3/294) each with lymphadenopathy and muscle weakness. Overall, 26.53% of dogs (79/294) showed at least one symptom compatible with the investigated vector-borne diseases, i.e., ehrlichiosis, anaplasmosis, babesiosis, borreliosis, and dirofilariosis. Further clinical findings included alopecia (9.18%, 27/294), nail overgrowth (2.43%, 7/294), purulent eye discharge (1.36%, 4/294), lameness (0.68%, 2/294), and cough (0.34%, 1/294).

**Table 1:** Seroprevalence of *Anaplasma* spp., *Ehrlichia* spp., *Borrelia burgdorferi* s.l., *Babesia* spp. and prevalence of *Dirofilaria immitis* antigen in dogs from Costa Rica.

**Province**	**No. of dogs sampled**	***Ehrlichia* spp.^a^**	***Anaplasma* spp.^a^**	***B. burgdorferi* s.l.^a^**	***Babesia* spp.^b^ [borderline samples]**	***D. immitis*^a^**
Alajuela	21	28.57% (6/21)	9.52% (2/21)	0.00% (0/21)	9.52% (2/21)	14.29% (3/21)
Cartago	22	13.63% (3/22)	4.55% (1/22)	0.00% (0/22)	0.00% (0/22)	0.00% (0/22)
Guanacaste	61	65.57% (40/61)	19.67% (12/61)	0.00% (0/61)	57.38% (35/61) [4.92% (3/61)]	0.00% (0/61)
Heredia	44	25.00% (11/44)	15.91% (7/44)	0.00% (0/44)	20.45% (9/44) [11.36% (5/44)]	0.00% (0/44)
Limón	47	29.78% (14/47)	10.64% (5/47)	0.00% (0/47)	12.77% (6/47) [10.64% (5/47)]	2.13% (1/47)
Puntarenas	73	52.05% (38/73)	16.48% (12/73)	1.37% (1/73)	20.55% (15/73) [9.59% [7/73)]	12.33% (9/73)
San José	26	15.38% (4/26)	0.00% (0/26)	0.00% (0/26)	3.85% (1/26)	0.00% (0/26)
Total	294	39.46% (116/294; 95% CI: 33.83–45.29%)	13.27% (39/294; 95% CI: 9.61 −17.69%)	0.34% (1/294; 95% CI: 0.01–1.88%)	23.13% (68/294; 95% CI: 18.43–28.38%) [6.80% (20/294; 95% CI: 4.20–10.31%)]	4.42% (13/294; 95% CI: 2.38–7.44%)

a As determined by rapid ELISA (SNAP®4DXPlus®, IDEXX Laboratories Inc.).

b As determined by microtitre plate ELISA (Babesia ELISA DOG, afosa GmbH).

*CI, confidence interval*.

### Seroprevalence of Rickettsiales and *Babesia* spp. and Effect on PCV

Overall, 45.24% (133/294, 95% CI: 39.45–51.11%) of dogs were seropositive for at least one of the tested vector-borne pathogens. Seroprevalence was highest for *Ehrlichia* spp. (39.46%, 116/294, 95% CI: 33.83–45.29%), followed by *Babesia* spp. (23.13%, 68/294, 95% CI: 18.43–28.38%). An additional 6.80% (20/294, 95% CI: 4.20–10.31%) of dogs showed a borderline *Babesia* ELISA test result. Seroprevalence of *Anaplasma* spp. as indicated by the rapid ELISA was 13.27% (39/294, 95% CI: 9.61–17.69%), and *Borrelia burgdorferi* s.l. antibodies were found in a single dog (0.34%, 95% CI: 0.01–1.88%). Seropositivity for more than one pathogen was observed in 23.13% of all dogs (68/294, 95% CI: 18.43–28.38%). Rates of co-exposure for the different pathogens are displayed in Table 2.

**Table 2:** Single and multiple exposure to vector-borne pathogens among 294 Costa Rican dogs as assessed by rapid ELISA (*Ehrlichia* spp., *Anaplasma* spp., *Borrelia burgdorferi* s.l. and *Dirofilaria immitis*), and microtitre plate ELISA (*Babesia* spp.^a^).

	**Seropositive/total**	**% Seropositive**	**95% CI**
*Ehrlichia* spp.	45/294	15.31	11.39–19.94
*Anaplasma* spp.	6/294	2.04	0.75–4.39
*Babesia* spp.^a^	9/294	3.06	1.41–5.73
*Ehrlichia* + *Anaplasma* spp.	4/294	1.36	0.04–3.45
*Ehrlichia* + *Babesia* spp.^a^	33/294	11.22	7.85–15.40
*Ehrlichia* spp. + *Dirofilaria immitis*	6/294	2.04	0.75–4.39
*Anaplasma + Babesia* spp.^a^	1/294	0.34	0.01–1.88
*Ehrlichia + Anaplasma + Babesia* spp.^a^	17/294	5.78	3.40–9.10
*Ehrlichia* spp. + *Dirofilaria immitis + Borrelia burgdorferi* s.l.	1/294	0.34	0.01–1.88
*Ehrlichia + Anaplasma + Babesia* spp.^a^ + *Dirofilaria immitis*	6/294	2.04	0.75–4.39

a Excluding borderline Babesia-ELISA test results.

*CI, confidence interval*.

Because retesting of dogs with a borderline *Babesia* spp. ELISA result after 4–6 weeks, as recommended by the test manufacturer, was not possible in this study, sera with borderline test results were excluded from further analyses. In addition, no information on breed was available for one dog, resulting in a final sample size of *N* = 273 for statistical analyses. Generalized linear mixed models indicated that seroprevalence for *Ehrlichia, Anaplasma*, and *Babesia* spp. was significantly associated (Table 3). Specifically, the models estimated that *Babesia*-seropositive dogs had 13.69 times higher odds of also being *Ehrlichia*-seropositive and 7.91 times higher odds of also being *Anaplasma*-seropositive (Table 3, *P* < 0.001). In addition, age and breed were significant predictors of *Ehrlichia*-seropositivity, with older dogs and mongrels having a higher probability of being seropositive (GLMM, Table 3, *P* = 0.043 and *P* = 0.018, respectively). Regarding *Anaplasma*- and *Babesia*-seropositivity, neither a significant effect of age nor of breed was observed.

**Table 3:** Results of binomial GLMMs testing the influence of different predictor variables on the probability of testing seropositive for *Ehrlichia* spp. (Model A), *Anaplasma* spp. (Model B) and *Babesia* spp. (Model C), amongst 273 dogs from Costa Rica.

	**Model A:** ***Ehrlichia*** **spp. seropositive**					**Model B:** ***Anaplasma*** **spp. seropositive**					**Model C:** ***Babesia*** **spp. seropositive**				
	**Estimate**	**SE**	***z***	***P***	**OR**	**Estimate**	**SE**	***z***	***P***	**OR**	**Estimate**	**SE**	***z***	***P***	**OR**
Intercept	−1.87	0.61	−3.05	**0.002**		−3.26	0.68	−4.79	**<0.001**		−3.78	0.82	−4.63	**<0.001**	
Sex (ref. male)	−0.11	0.35	−0.31	0.756	0.90	0.40	0.43	0.93	0.355	1.49	−0.42	0.43	−0.99	0.323	0.66
Age	0.11	0.05	2.03	**0.043**	1.12	0.00	0.07	0.07	0.945	1.00	0.06	0.07	0.91	0.362	1.06
Breed (ref. “with breed”)	−1.13	0.48	−2.38	**0.018**	0.32	−0.57	0.60	−0.94	0.349	0.57	−0.73	0.57	−1.28	0.201	0.48
*Anaplasma* spp. seropositive	1.22	0.64	1.91	0.056	3.37	–	–	–	–	–	2.11	0.57	3.72	**<0.001**	8.22
*Babesia* spp. seropositive	2.62	0.45	5.84	**<0.001**	13.69	2.07	0.53	3.90	**<0.001**	7.91	–	–	–	–	–
*Ehrlichia* spp. seropositive	–	–	–	–	–	0.85	0.55	1.56	0.119	2.34	2.56	0.45	5.71	**<0.001**	12.95
**Area**^**a**^															
East coast–city	0.28	0.89	0.31	0.998	1.32	0.11	0.81	0.14	0.999	1.12	0.67	1.08	0.62	0.972	1.95
West coast–city	1.88	0.82	2.30	0.142	6.56	0.83	0.71	1.18	0.761	2.30	0.69	1.02	0.67	0.962	1.99
Rural-high^b^-city	−0.53	0.81	−0.66	0.965	0.59	0.19	0.74	0.25	0.999	1.21	0.26	1.13	0.23	0.999	1.29
Rural-low^c^-city	0.24	0.70	0.34	0.997	1.27	−0.80	0.64	−1.24	0.724	0.45	1.23	0.96	1.28	0.699	3.42
West coast–east coast	1.60	1.05	1.53	0.535	4.97	0.72	0.84	0.87	0.908	2.06	0.02	1.17	0.02	1.000	1.02
Rural-high^b^-east coast	−0.81	0.96	−0.85	0.914	0.44	0.08	0.83	0.09	1.000	1.08	−0.41	1.22	−0.34	0.997	0.66
Rural-low^c^-east coast	−0.04	0.88	−0.05	1.000	0.96	−0.91	0.74	−1.23	0.730	0.40	0.56	1.08	0.52	0.985	1.75
Rural-high^b^-west coast	−2.41	0.97	−2.49	0.091	0.09	−0.65	0.78	−0.83	0.921	0.52	−0.43	1.22	−0.35	0.997	0.65
Rural-low^c^-west coast	−1.64	0.87	−1.90	0.315	0.19	−1.63	0.64	−2.54	0.080	0.20	0.54	1.07	0.51	0.987	1.72
Rural-low^c^-rural high^b^	0.77	0.81	0.96	0.871	2.16	−0.98	0.69	−1.44	0.601	0.37	0.97	1.03	0.94	0.880	2.64

Full models were significantly different from null models containing only the random factor “sampling location” [likelihood ratio test, χ^2^ = 79.8, df = 9, P < 0.001 (Model A), χ^2^ = 46.8, df = 9, P < 0.001 (Model B) and χ^2^ = 83.8, df = 9, P < 0.001 (Model C), respectively]. Significant P-values (≤ 0.05) are printed in bold.

a Multiple comparisons between levels of the factor “Area” were performed using Tukey contrasts with single-step p-value adjustment.

b ≥1,000 m asl

c < 1,000 m asl

*SE, Standard error; OR, Odds ratio*.

**Figure 1:**
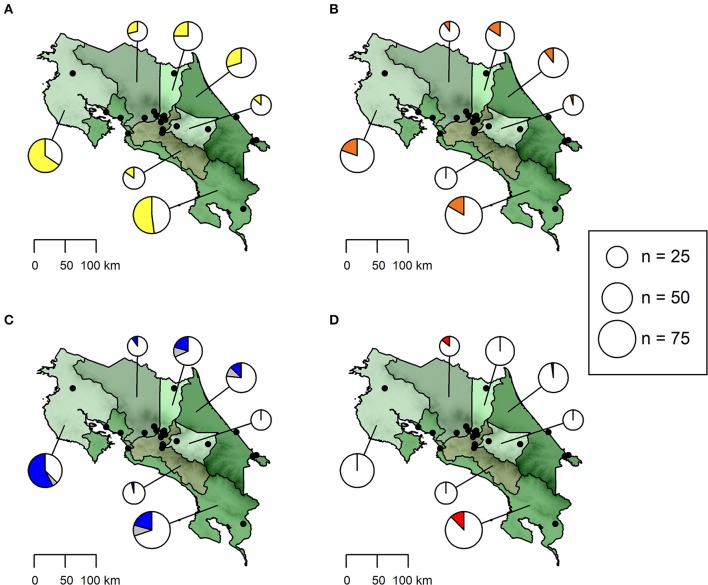
Seroprevalence of antibodies against **(A)** *Ehrlichia* spp. (yellow), **(B)** *Anaplasma* spp. (orange) and **(C)** *Babesia* spp. (blue) as well as **(D)** *Dirofilaria immitis* antigen (red) in dogs from Costa Rica. The size of pie charts corresponds to the number of dogs sampled per province. Sampling locations are shown as black dots. The proportion of samples with a doubtful *Babesia* spp. test result is indicated in gray.

On province level, seroprevalence rates were highest in Guanacaste (*Ehrlichia* spp.: 65.57%, *Babesia* spp.: 57.38%, *Anaplasma* spp.: 19.67%) and lowest in Cartago (*Ehrlichia* spp.: 13.63%, *Babesia* spp.: 0.00%, *Anaplasma* spp.: 4.55%) (Table 1, Figure 1). However, no statistically significant differences in seroprevalence were found between dogs sampled in rural areas of high or low altitude, eastern or western coastal areas or cities (GLMMs, Table 3).

**Figure 2:**
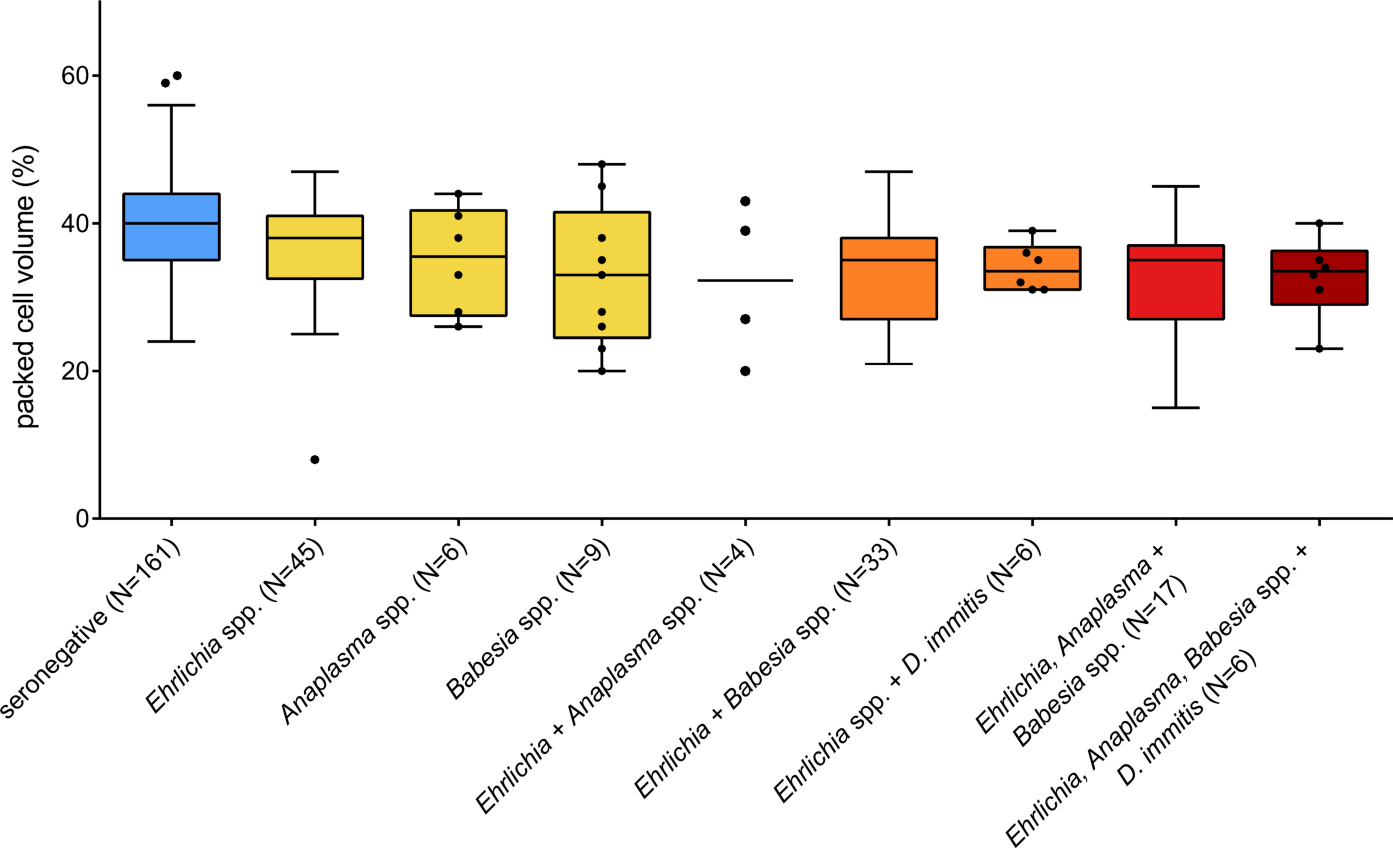
Packed cell volume of dogs seropositive for different vector-borne pathogens in Costa Rica. Only one animal was seropositive for *Anaplasma* and *Babesia* spp. and was not plotted. Boxes extend from the 25th to the 75th percentile, with a line at the median and whiskers extending to 1.5 the interquartile range or up to the maximum/minimum value. Individual data points are shown for *N* < 10.

No significant difference was observed regarding the proportion of animals showing clinical signs compatible with vector-borne disease when comparing animals seropositive for at least one of the tested vector-borne pathogens to seronegative animals (χ^2^ = 2.76, df = 1, *P* = 0.097). However, an effect of seropositivity on PCV was found for *Babesia* spp., *Ehrlichia* spp., and *Anaplasma* spp., and the interaction between *Babesia* spp. and *Ehrlichia* spp. was also statistically significant (LMM, Table 4). *Babesia*-seropositive dogs showed on average 6.3% lower PCV values than seronegative dogs (*P* = 0.003). The effect was less pronounced for *Ehrlichia*- and *Anaplasma*-seropositive dogs, which showed approximately 2.9% lower PCV values on average than seronegative animals (*P* = 0.016 and *P* = 0.034, respectively). Being seropositive for both *Babesia* spp. and *Ehrlichia* spp. led to a less pronounced reduction of PCV than expected if the effect had been additive, namely a 4.1% reduction in PCV on average, compared to seronegative dogs. PCV values of seronegative and seropositive dogs are displayed in Figure 2.

**Table 4:** Results of LMM testing the influence of animal sex, age, and seropositivity for *Ehrlichia* spp., *Anaplasma* spp. and *Babesia* spp. antibodies as well as *D. immitis* antigen on packed cell volume of 273 dogs from Costa Rica.

	**Estimate**	**SE**	**df**	***t***	***P***
Intercept	39.61	1.23	26.24	32.11	**<0.001**
Sex (ref. male)	0.95	0.84	256.88	1.14	0.257
Age	−0.13	0.13	255.44	−0.99	0.321
*Ehrlichia*-seropositive	−2.96	1.22	257.87	−2.42	**0.016**
*Anaplasma*-seropositive	−2.91	1.37	252.81	−2.13	**0.034**
*Babesia*-seropositive	−6.30	2.11	248.32	−2.98	**0.003**
*D. immitis* antigen-positive	−2.62	2.34	261.36	−1.12	0.262
*Babesia*-seropositive: *Ehrlichia*-seropositive	5.16	2.47	246.48	2.09	**0.037**

*The full model was significantly different from a null model containing only the random factor “Location of sampling“ (likelihood ratio test, χ^2^ = 38.91, df = 7, P < 0.001). Significant P-values are printed in bold. SE, Standard error*.

### Current Infections

In the blood samples of 51.72% (60/116, 95% CI: 42.26–61.10%) of *Ehrlichia*-seropositive dogs, *E. canis* DNA was detected by conventional PCR and/or qPCR. In contrast, neither *E. ewingii* nor *E. chaffeensis* were detected in any sample. Only 33.33% (20/60, 95% CI: 21.69–46.69%) of *E. canis*-positive dogs showed clinical symptoms, namely pale mucous membranes, apathy, fever, epistaxis or a combination of these.

Furthermore, 12.82% (5/39, 95% CI: 4.30–27.43%) of *Anaplasma*-seropositive dogs were also PCR-positive. Four of these five animals were co-infected with *A. platys* and *A. phagocytophilum*, while one animal was infected with *A. phagocytophilum* only. Among the 32 animals tested by PCR for *Anaplasma* spp. as well as *E. canis* based on a positive serological result, 15 (46.88%, 95% CI: 29.09–65.26%) were mono-infected with *E. canis*, two (6.25%, 95% CI: 0.77–20.81%) were co-infected with *A. platys* and *A. phagocytophilum* and one dog (3.13%, 95% CI: 0.08–16.22%) was infected with all three pathogens. Only the triple-infected dog showed pale mucous membranes in the clinical examination, while no symptoms were noted in the remaining *Anaplasma*-infected animals.

Of the 68 *Babesia*-seropositive dogs, only one (1.47%, 95% CI: 0.04–7.92%) was positive for *B. vogeli* in the PCR (100% sequence identity [ID], 99% query cover [QC]), but did not show any clinical symptoms of babesiosis, whereas *Hepatozoon canis* DNA was amplified from a second, asymptomatic dog (99% ID, 98% QC). The *H. canis-*infected dog was also seropositive for *Ehrlichia* spp., but negative in the *Ehrlichia* PCR. The *B. vogeli*-infected dog was seronegative for all other pathogens tested, thus, no further PCRs were carried out.

*Dirofilaria immitis* antigen was detected only in dogs from the provinces Alajuela, Limón and Puntarenas (Table 1, Figure 1), with an overall prevalence of 4.42% (13/294, 95% CI: 2.38–7.44%). Microfilariae were detected in buffy coat of 11 dogs, three of which were also positive for *D. immitis* antigen in the rapid ELISA and yielded a positive *D. immitis* PCR result (96% ID, 98% QC). *Acanthocheilonema reconditum* DNA (99% ID, 97% QC) was amplified from the blood samples of three further microfilaremic dogs, which were tested negative for *D. immitis* antigen in the rapid ELISA. For the remaining five dogs, the filarial species could not be identified, as no amplicon resulted from the PCR.

## Discussion

Canine vector-borne diseases, including important zoonoses, are widespread in Central America. In the present study, exposure to at least one of five tested pathogens was detected in 45.24% of the 294 tested dogs, while multiple exposure was demonstrated in 23.13%. A significant association between seropositivity for *Ehrlichia, Anaplasma* and *Babesia* spp. was shown. This may be due to the fact that *E. canis* and *A. platys* as well as *B. vogeli* and *B. gibsoni* share a common vector, namely the brown dog tick, *R. sanguineus* s.l., which is the most common tick species parasitizing dogs in Central America (5–7). Furthermore, experimental infections have shown that concurrent *Ehrlichia*-infection intensifies the humoral immune response to *A. platys* in dogs, resulting in a more persistent *A. platys* infection (35). Similar immune-mediated interactions could apply to *Babesia/Ehrlichia* or *Babesia/Anaplasma* co-infections, however, no experimental data on these combinations are available to date.

Seroprevalence of *Ehrlichia* spp. was almost 40%, which is comparable to previous studies conducted in Costa Rica (7, 11) and Mexico (36), whereas a considerably higher seroprevalence of more than 60% was detected in the neighboring country of Nicaragua (10). *Ehrlichia*-seropositive dogs were found in all seven Costa Rican provinces, and no statistically significant differences between different sampling locations (urban areas, high/low elevation rural areas or coastal areas) were found. Nevertheless, the highest prevalences were detected in the provinces of Guanacaste and Puntarenas, bordering the Pacific Coast, similar to the pattern reported by Montenegro et al. (11). Older dogs as well as mongrels had a higher probability of being *Ehrlichia*-seropositive, which also confirms previous findings (7, 11).

Current *E. canis* infections, as defined by amplification of *E. canis* DNA by PCR, were detected in 51.72% of *Ehrlichia*-seropositive dogs. The high rate of current *E. canis* infections in asymptomatic dogs is concerning, if the pathogen might also infect humans. Recently, anti-*Ehrlichia* spp. antibodies have been found in 35% of 280 human blood donor samples examined in Costa Rica, with 3.5% of samples containing DNA of a novel *E. canis* genotype (37). In contrast, *E. chaffeensis* and *E. ewingii*, which possess higher zoonotic potential, were not detected in the present study, nor in the mentioned blood donor study (37). *E. chaffeensis* DNA was isolated from symptomatic human patients in Costa Rica (38), but the pathogen has neither been found in dogs nor in ticks in Central America so far.

Regarding *Anaplasma* spp., the present study may indicate an increasing seroprevalence of this genus in Costa Rica. In 2011, Bonilla et al. (13) detected 2.7% *Anaplasma*-seropositive animals among 408 sampled dogs, with regional prevalences up to 6.5%. A similar study from 2011 to 2012, which tested 314 Costa Rican dogs with the same method as in the present study, detected a country-wide *Anaplasma* spp. seroprevalence of 6.5%, with the highest value in the province of Guanacaste (16.2%) (11). In the present study, based on a comparable sample size, *Anaplasma*-seropositive dogs were detected in six of the seven Costa Rican provinces, overall *Anaplasma* spp. seroprevalence was 13.27%, and reached 19.27% in Guanacaste. Thus, canine anaplasmosis may constitute an emerging infection in Costa Rica. Nevertheless, the detected differences might also be due to methodological reasons, since the 2011/12 survey by Bonilla et al. (13) used a different serological test. Furthermore, although all three surveys covered the seven provinces of Costa Rica, actual sampling locations differed. For example, the present survey covered more coastal regions, while the 2011/12 survey mainly focused on the Greater Metropolitan area (as described in 7). Thus, local variation in seroprevalence may also underlie the detected differences, as well as further factors such as the age or breed composition of the study populations.

Among *Anaplasma*-seropositive dogs, *A. platys* as well as *A. phagocytophilum* were detected at almost the same frequency, and predominantly as co-infections. A similar infection pattern was found in dogs from Nicaragua (10). In Central America, ixodid ticks, which are the usual vectors for *A. phagocytophilum*, are rather rare as canine parasites (5, 39). However, a low prevalence of *A. phagocytophilum* has been found in *R. sanguineus* s.l. collected from dogs in Costa Rica (12). Nevertheless, it remains unknown whether *R. sanguineus* s.l. might be implicated in the transmission of zoonotic *A. phagyocytophilum* in Central America.

The present study contains the first large-scale serological survey of canine babesiosis in Central America, and demonstrates the presence of the pathogen in six of seven Costa Rican provinces, with an overall seroprevalence of more than 20%. Previous studies on *Babesia* spp. in dogs from Costa Rica used a PCR-based approach, thus detecting only current infections. Wei et al. (15) demonstrated current *Babesia*-infections by quantitative real-time PCR (qPCR) in 10 of 40 dogs sampled in northwestern Costa Rica. In Nicaragua, a similar infection rate of 26% was found in a sample of 39 dogs (16). Both *B. vogeli*- and *B. gibsoni*-infections were detected in these studies. In contrast, a lower prevalence of 8% was found among 146 Costa Rican dogs tested by conventional PCR (14). In the present study, *B. vogeli* DNA was only amplified from one seropositive dog, while *B. gibsoni* DNA was not detected. The discrepancies in infection rates between these studies might be due to geographical differences in *Babesia* prevalence within Costa Rica, as well as to a higher sensitivity of qPCRs compared to conventional PCRs, or to sensitivity differences related to the different target genes. Chronic infections with *B. vogeli* and *B. gibsoni* are commonly associated with very low parasitemia, and it is additionally recommended to use capillary rather than venous blood or buffy coat preparations for diagnosis (23, 40). Low parasitemias and the fact that venous blood was used in this study may have negatively affected PCR sensitivity. *Babesia*-seropositive dogs, as well as *Ehrlichia-* and *Anaplasma*-seropositive dogs, had a significantly lower PCV as compared to seronegative dogs, as shown in previous studies [e.g., (10, 14)].This also indicates that the rate of current infections among *Babesia*-seropositive dogs may have actually been higher than detected. Alternatively, the lower PCV values in *Babesia*-seropositive dogs may be a sign of immune-mediated hemolytic anemia, a complication of canine babesiosis due to the production of anti-erythrocyte antibodies, which may persist even after the infection has been cleared (41). Furthermore, only blood of seropositive dogs was tested for current infections, thus, we may have missed current infections in dogs which had not seroconverted yet.

Although not specifically targeted in this study, *H. canis* DNA was detected in one dog, confirming previous reports from Costa Rica (14). Both *R. sanguineus* s.l. and *Amblyomma ovale* may act as vectors for this apicomplexan parasite and both tick species occur in Central America (5, 6). *Hepatozoon canis* mostly causes moderate or asymptomatic infections with low parasitemia in dogs and is not considered a zoonotic pathogen (42). However, severe clinical signs may occur in cases of canine hepatozoonosis characterized by a high level of parasitemia, and co-infections with other pathogens are common, complicating the clinical picture (42).

*Dirofilaria immitis* infections in Costa Rican dogs have so far mainly been found in the provinces of Guanacaste and Puntarenas, bordering the Pacific Coast (11, 15, 17). The present study confirms these results, as the majority of *D. immitis*-positive dogs (9/13 infected animals) were from the province of Puntarenas. The three *D. immitis*-positive dogs in the central Costa Rican province of Alajuela may have been translocated from a coastal region. Alternatively, this might indicate a geographic spread of the parasite to central regions of Costa Rica, which needs to be confirmed in future studies.

In addition to *D. immitis, A. reconditum*, which is transmitted by fleas, was identified in three microfilaremic dogs from the eastern parts of Costa Rica (provinces Heredia and Limón). Although flea infestation is common in dogs all over Costa Rica, the regional presence of *A. reconditum* confirms findings by Rojas et al. (17), who detected a high prevalence of *A. reconditum* in the province of Limón. Thus, *A. reconditum* needs to be considered as a differential diagnosis to dirofilariosis if microfilaria are observed in these areas. *Acanthocheilonema reconditum* is considered as less pathogenic than *D. immitis*, and is also of less zoonotic importance (43).

As in previous studies from Central America and Mexico (10, 11, 36), *B. burgdorferi* s.l. seroprevalence was very low. Here, only one seropositive dog was found, and it cannot be excluded that this dog had a travel history and got infected outside of Central America. Furthermore, it should be borne in mind that the positive predictive value of a diagnostic test, i.e., the number of true positives among all positive test results, is influenced by the prevalence of the pathogen as well as by the test's sensitivity and specificity. Since the prevalence of *B. burgdorferi* s.l. was very low, the resulting positive predictive value is also low (21.6%), thus, positive test results for *B. burgdorferi* s.l. in this region should be treated with caution, as the probability of false-positive results is high.

Clinical symptoms compatible with the investigated vector-borne diseases were noted in more than 25% of the studied dogs. However, apart from a lower PCV in *Ehrlichia*-, *Anaplasma-*, and *Babesia*-seropositive animals, no statistically significant association between seropositivity and clinical signs was found. Many of these symptoms, such as anorexia and apathy, are rather unspecific. They occurred equally often in seronegative animals, possibly due to other infectious or non-infectious causes, whereas severe symptoms of vector-borne diseases, e.g., petechial bleeding, were only noted in very few dogs. Furthermore, the incubation period following a tick bite for anaplasmosis and babesiosis is shorter (~1 week) than the time to seroconversion (~2 weeks) (23) and may thus further explain the missing association.

## Conclusions

This study demonstrated high seroprevalences of several canine vector-borne pathogens in Costa Rica, with a possible rise of *Anaplasma* spp. infections as compared to previous surveys. In addition, *Babesia*-seroprevalence was assessed for the first time in Costa Rican dogs, revealing exposure of more than 20% of dogs. Although most animals were asymptomatic, a significant effect of *Ehrlichia, Anaplasma* and *Babesia* seropositivity on PCV was found. In addition, chronically infected dogs may constitute a reservoir of human infection in the case of zoonotic pathogens, such as *A. phagocytophilum* and *D. immitis*. Thus, protection of dogs from disease-transmitting vectors is recommended from an animal welfare as well as public health perspective.

## Data Availability

All datasets supporting the conclusions of the study are included in the manuscript.

## Ethics Statement

Analyzed blood samples represent surplus from diagnostic blood samples of dogs that were presented at veterinary clinics for varying reasons. Consent of the dog owners to use surplus samples for further analyses was obtained.

## Author Contributions

CS and VM designed and coordinated the study. VM collected the blood samples. VM, SS, MG, NP, JB and AS performed laboratory analyses. AS performed the statistical analyses and drafted the manuscript. All authors participated in data analysis and interpretation. All authors read and approved the final version of the manuscript.

### Conflict of Interest Statement

MG, NP, and JB are currently employed by IDEXX Laboratories. Study data collection and interpretation is completely independent from the company's opinion. The remaining authors declare that the research was conducted in the absence of any commercial or financial relationships that could be construed as a potential conflict of interest.
